# Pathogenic Variants Associated with Epigenetic Control and the NOTCH Pathway Are Frequent in Classic Hodgkin Lymphoma

**DOI:** 10.3390/ijms25052457

**Published:** 2024-02-20

**Authors:** Antonio Santisteban-Espejo, Irene Bernal-Florindo, Pedro Montero-Pavon, Jose Perez-Requena, Lidia Atienza-Cuevas, Maria del Carmen Fernandez-Valle, Ana Villalba-Fernandez, Marcial Garcia-Rojo

**Affiliations:** 1 Department of Pathology, Puerta del Mar University Hospital, 11009 Cadiz, Spain; jose.perez.sspa@juntadeandalucia.es (J.P.-R.); anavillalbafernandez@hotmail.com (A.V.-F.); 2 Department of Medicine and Surgery, Faculty of Medicine, University of Cadiz, 11003 Cadiz, Spain; 3 Institute of Research and Innovation in Biomedical Sciences of the Province of Cadiz (INiBICA), 11009 Cadiz, Spain; iriberni@hotmail.com (I.B.-F.); marcial.garcia.sspa@juntadeandalucia.es (M.G.-R.); 4 Department of Pathology, Jerez de la Frontera University Hospital, 11407 Cadiz, Spain; pmonteropavon@gmail.com; 5 Department of Hematology and Hemotherapy, Puerta del Mar University Hospital, 11009 Cadiz, Spain; macafeva@hotmail.com

**Keywords:** classic Hodgkin lymphoma, clonal evolution, next-generation sequencing, epigenetics, personalized therapies

## Abstract

Classic Hodgkin lymphoma (cHL) constitutes a B-cell neoplasm derived from germinal center lymphocytes. Despite high cure rates (80–90%) obtained with the current multiagent protocols, a significant proportion of cHL patients experience recurrences, characterized by a lower sensitivity to second-line treatments. The genomic background of chemorefractory cHL is still poorly understood, limiting personalized treatment strategies based on molecular features. In this study, using a targeted next-generation sequencing (NGS) panel specifically designed for cHL research, we compared chemosensitive and chemorefractory diagnostic tissue samples of cHL patients. Furthermore, we longitudinally examined paired diagnosis–relapsesamples of chemorefractory cHL in order to define patterns of dynamic evolution and clonal selection. Pathogenic variants in NOTCH1 and NOTCH2 genes frequently arise in cHL. Mutations in genes associated with epigenetic regulation (CREBBP and EP300) are particularly frequent in relapsed/refractory cHL. The appearance of novel clones characterized by mutations previously not identified at diagnosis is a common feature in cHL cases showing chemoresistance to frontline treatments. Our results expand current molecular and pathogenic knowledge of cHL and support the performance of molecular studies in cHL prior to the initiation of first-line therapies.

## 1. Introduction

Classic Hodgkin lymphoma (cHL) is a B-cell neoplasm derived from germinal center lymphocytes at different stages of development [1,2]. With current multiagent treatment protocols, based on the combination of chemotherapy, radiotherapy, anti-CD30 conjugates and immunotherapy, the majority of patients (80–90%) achieve a complete metabolic response without recurrences of the disease [3,4]. However, there is still a significant fraction (10–20%) of cHL patients who are either primary refractory to conventional treatment or relapse after achieving a previous response. The reasons for this differential therapeutic sensitivity are poorly understood.

Consequently, the elucidation of the genomic events that could allow the early identification of high-risk patients constitutes an unmet need in cHL translational research. Previous works have identified genomic aberrations affecting Nuclear Factor Kappa B (NFKB) [5], Janus kinase/signal transducer and activator of transcription (JAK-STAT) [6] and, recently, Hippo Yes-associated protein (YAP)/WW domain-containing transcription regulator 1 (TAZ) (Hippo /TAZ/YAP) [7] pathways as drivers of cHL pathogenesis. Particularly, mutations involving the TP53 gene and epigenetic control seem to frequently arise in relapsed/refractory cHL [8], but the scarcity of literature limits the generalization of conclusions. Targeted mutation analysis of cHL-associated genes detected by next-generation sequencing (NGS) could contribute to personalizing therapeutic decisions [9,10] and improving cure rates in cHL.

In this study, using a targeted NGS panel specifically designed for cHL research [11], we sequenced cHL tumor samples obtained at diagnosis from patients achieving a complete metabolic response and compared them with cHL tumor samples from relapsed/refractory cases. Furthermore, we analyzed paired tissue samples obtained at diagnosis and recurrences to describe the pattern of mutation selection and clonal evolution during the course of the disease.

Our results demonstrated that pathogenic variants in NOTCH1, NOTCH2 and epigenetic regulators (CREBBP, EP300) arise particularly frequently in chemorefractory patients. Furthermore, the appearance of novel clones harboring mutations initially not detected at diagnosis constitutes a common event in cases showing chemoresistance. These data support the performance of mutational analyses in newly diagnosed cHL patients prior to deciding the frontline treatment, and they expand the current biological knowledge of the mutational spectrum of the disease.

## 2. Results

### 2.1. Characteristics of the Patients

The main clinical and histopathological characteristics of the patients in this study are summarized in Table 1. In our sample, 59.1% of the patients (13/22) were female, and the most frequent histological subtype was nodular sclerosis cHL (77.3%). Epstein–Barr virus (EBV) latent membrane protein-1 (LMP-1) was detected in 6 out 22 cases (27.3%). At diagnosis, 10 patients (45.5%) presented in initial stages, while 12 out of 22 (54.4%) presented in advanced stages (III–IV). Bulky disease and B symptoms were present at diagnosis in 13.6% and 63.6% of the patients, respectively. In total, 10 out of 22 cHL patients (45.5%) were primarily refractory to first-line treatment or relapsed after achieving a complete metabolic response.

### 2.2. Next-Generation Sequencing of Classic Hodgkin Lymphoma Reveals Intratumoral Heterogeneity

All the samples analyzed showed pathogenic variants in the genes defined in the sequencing panel. The mean allele frequency was 5.1% (2.2–33.5%) and the mean coverage was 1691.5× (101×–2000×) (Figures S1 and S2). Using an NGS panel for B-cell neoplasms, specifically designed for this project (Table S1) [11] from an initial set of 38,384 variants, and after applying a stringent bioinformatics pipeline (see Section 4.4), we identified 226 pathogenic variants in the 32 cHL samples evaluated (Figure S3, Table S2). Thus, 58.9% of the genomic variants originally found were discarded after filtering and variant calling. The identification of recurrent mutations previously described in cHL [5,6,8], the concordance of variants obtained in paired samples (R^2^ = 0.8, Figure S4) and the strict variant calling process attest to the robustness of the analysis and the data obtained.

**Figure 1:**
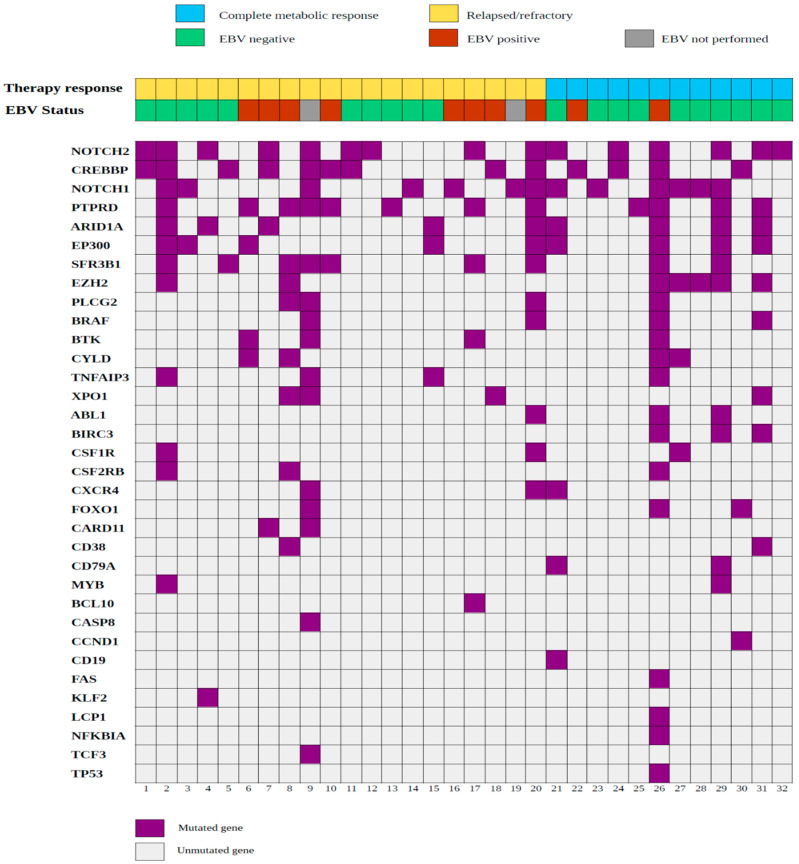
Heat map of nonsynonymous somatic mutations identified in all the cHL cases analyzed. Each column represents a cHL case, and each row represents a gene.

The pathogenic variants identified in all the cases are shown in Figure 1. Mutations affected genes such as NOTCH1, NOTCH2, PTPRD, CREBBP and ARID1A, which are involved in B-cell development, signaling control and epigenetic regulation [12]. The most frequent mutations implicated variants in the NOTCH1 (16.4%) and NOTCH2 (11.9%) genes. Deregulation of the NOTCH pathway is a well-known event in cHL pathogenesis [13,14,15,16]. Moreover, variants in the PTPRD (7.9%), CREBBP (7.1%) and ARID1A (6.6%) genes that were previously reported in NGS studies of cHL [5,8] were also identified.

The absence in our NGS panel of the SOCS1 gene, an important suppressor of cytokine signaling involved in the control of the JAK-STAT pathway in cHL, precluded the identification of its mutational frequency in this study. It is also important to note that the frequency of STAT6 mutations in our cohort contrasted with data previously published on the mutational landscape of cHL, such as the works by Spina et al. [6]. This fact could be related, firstly, to the small sample; probably, a larger cohort would reveal a higher frequency of STAT6 mutations.

Genomic heterogeneity in cHL could also explain this finding in part; mutations affecting the regulation of the JAK-STAT pathway were reported in our study, involving different members implicated in this cellular pathway, such as pathogenic variants in the gene coding for phosphatases PTPRD (33.0%), and other drivers of this route, such as the genes coding for CSF2RB (8.8%) and TNFAIP3 (8.8%)

Overall, these results suggest that molecular alterations in cHL cooperate in different patterns to promote a disease phenotype differing significantly from non-Hodgkin lymphomas (NHLs) and acute leukemia, in which recurrent genomic alterations have been described and define well-established diagnostic and prognostic subgroups [17,18,19]. Furthermore, they indicate that in cHL, pathogenic variants in genes involved in the NOTCH, JAK-STAT (PTPRD) and epigenetic regulation (CREBBP, EP300, ARID1A) pathways arise frequently and explain, in part, the intratumoral heterogeneity that characterizes the disease.

### 2.3. Mutational Landscape of cHL Patients Achieving a Complete Metabolic Response after Induction with Adriamycin, Bleomycin, Vinblastine and Dacarbazine (ABVD)

Chemosensitive cHL cases had a lower mean allele frequency (mean allele frequency: 3.7%, range: 2.2–15.5%) than chemorefractory cHL cases (mean allele frequency: 6.2%, range: 12.2–33.5%). Coverage parameters did not differ between samples from patients achieving a complete metabolic response after induction (mean coverage: 1747.8×, range: 101×–2000×) and those from patients with chemorefractory cHL (mean coverage: 1635.7×, range: 113×–2000×).

**Figure 2:**
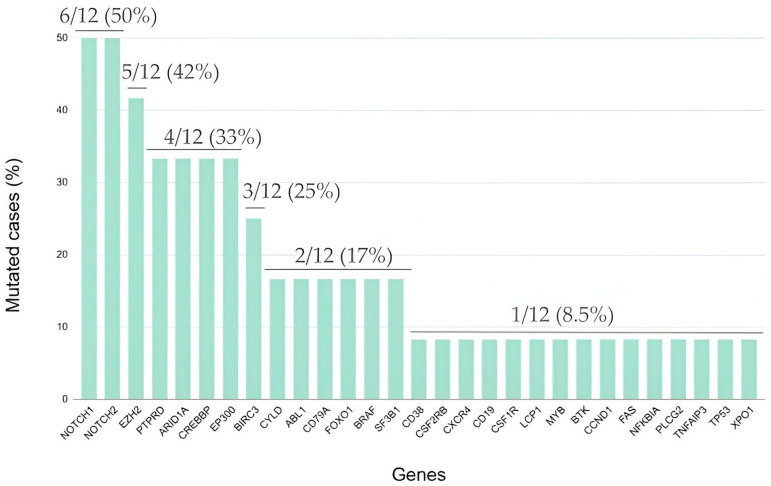
Mutational landscape of classic Hodgkin lymphoma (cHL) patients with a complete metabolic response after induction chemotherapy.

**Figure 3:**
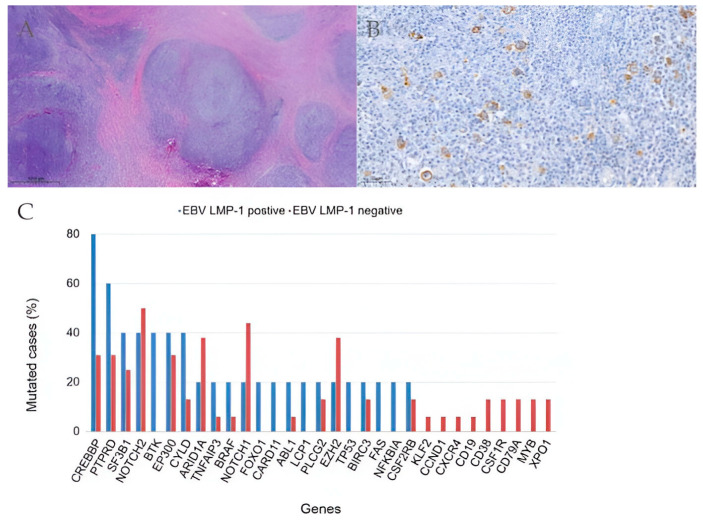
Histopathology of classic Hodgkin lymphoma (cHL) and expression of Epstein–Barr virus latent membrane protein-1 (EBV LMP-1). (**A**) Hematoxylin–eosin (H&E) staining showing fibrotic bands that delimitate a nodular pattern in a case of nodular sclerosis cHL (magnification 4×). (**B**) Immunohistochemistry for EBV LMP-1 shows a cytoplasmic and membranous staining in tumoral Hodgkin and Reed–Sternberg (HRS) cells (magnification 20×). Positivity for LMP-1 is restricted to cells that are cytologically identifiable as part of the tumor clone, i.e., HRS cells. Cells in the inflammatory background are shown as negative for LMP-1 expression, following the established recommendations for the interpretation of this marker [20]. (**C**) Pathogenic variants identified in EBV LMP-1-positive cHL (blue color) and EBV LMP-1-negative cHL (red color). Mutations were manually clustered to emphasize those genes in which LMP-1 was positive in ≥40% of cases.

Missense mutations of the NOTCH1 (50.0%), NOTCH2 (50.0%) and EZH2 (42.0%) genes most frequently arose in this group of patients (Figure 2). Extent of the disease (limited vs. advanced) and age (<45 years old vs. ≥45 years old) were not associated with the number of pathogenic variants identified. Interestingly, mutations in EBV-positive cHL differed from those in EBV-negative cHL cases. Most of the cases showing expression of EBV LMP-1 harbored somatic mutations in the CREBBP (80.0%), PTPRD (60.0%) and SF3B1 (40.0%) genes. However, in EBV-negative cHL, mutations mainly affected the XPO1, MYB, CD79A, CSF1R and CD38 genes (Figure 3).

### 2.4. Somatic Mutations in Genes Associated with Epigenetic Control Are Frequent in Relapsed/Refractory Classic Hodgkin Lymphoma

**Figure 4:**
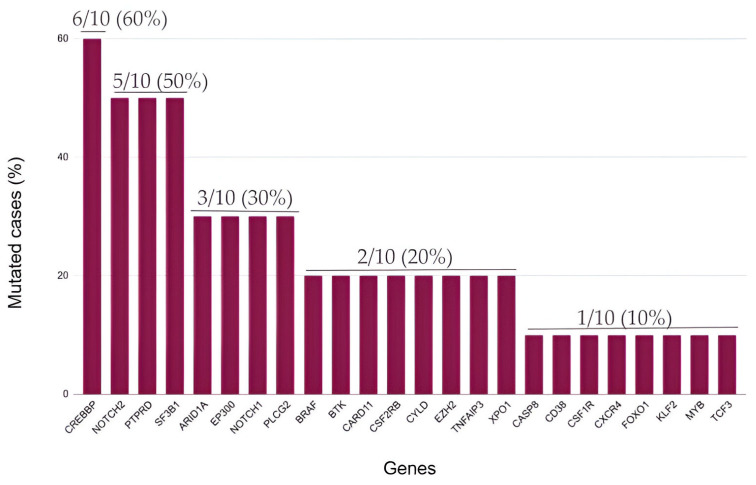
Mutations identified in relapsed/refractory classic Hodgkin lymphoma (cHL).

The most frequent mutations identified in relapsed/refractory cHL involved the CREBBP gene (60.0%) (Figure 4). Previously, in this subgroup of patients with an aggressive disease course and poor response to treatment, mutations in epigenetic regulators were reported as particularly frequent [8].

**Figure 5:**
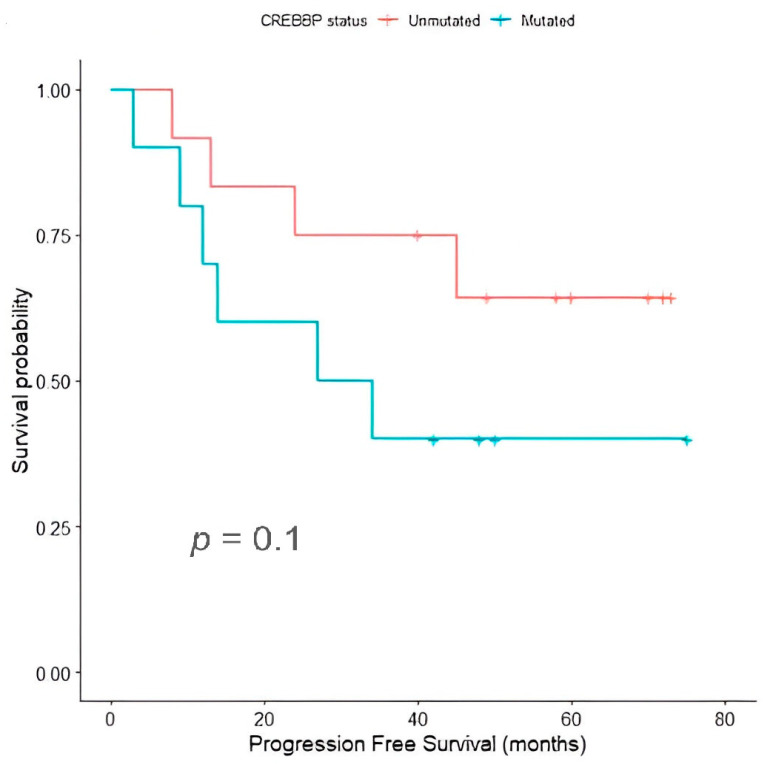
Progression-free survival distributions for classic Hodgkin lymphoma (cHL) patients depending on the mutational status of the CREBBP gene.

Differences in survival times did not reach statistical significance in cHL patients harboring CREBBP mutations (*p* = 0.1). However, cHL patients with mutated CREBBP presented a shorter PFS (mean PFS: 39.9 months; 95% CI: 21.4–58.4 months) in comparison with patients with wild-type CREBBP (mean PFS: 55.5 months; 95% CI: 41.1–69.9 months) (Figure 5). Together with NOTCH2 variants (50.0%) and mutations in PTPRD (50.0%), which is a gene coding for phosphatases involved in the JAK-STAT pathway, we found that somatic mutations of genes associated with epigenetic control other than CREBBP also frequently arose in this subgroup of patients, such as SF3B1 (50.0%), EP300 (30.0%) and ARID1A (30.0%). Overall, these data suggest an important role of epigenetic modifications in the development of chemoresistance in cHL.

**Figure 6:**
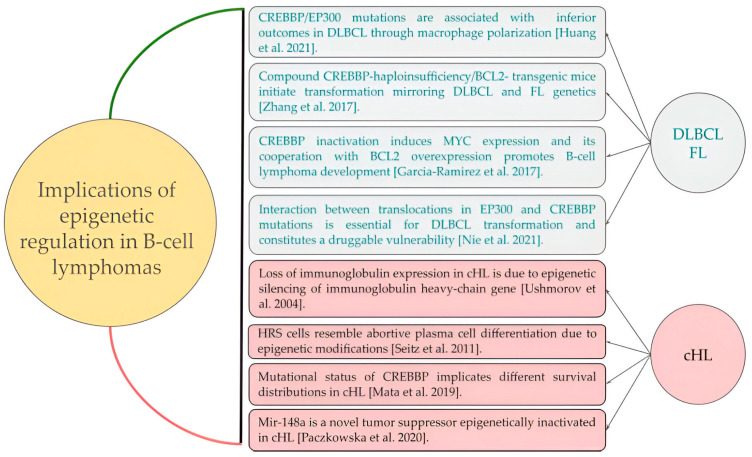
Contributions of epigenetic mechanisms to the pathogenesis and clinical outcomes of B-cell lymphomas (diffuse large B cell lymphoma, follicular lymphoma and classic Hodgkin lymphoma). DLBCL, diffuse large B cell lymphoma; FL, follicular lymphoma; cHL, classic Hodgkin lymphoma. Huang et al. 2021 [23], Zhang et al. 2017 [27], Garcia-Ramirez et al. 2017 [28], Nie et al. 2021 [24], Ushmorov et al. 2004 [22], Seitz et al. 2011 [29], Mata et al. 2019 [8], Paczkowska et al. 2020 [30].

Down-regulation of B-cell-specific transcription factors [21] and the immunoglobulin heavy-chain gene [22] by means of epigenetic silencing play an important role in the pathogenesis of cHL. As previously reported for diffuse large B-cell lymphoma (DLBCL) [23,24] and follicular lymphoma (FL) [25,26], pathogenic variants involving the CREBBP and EP300 genes seem to arise particularly frequently in patients with cHL showing chemorefractory disease (Figure 6).

### 2.5. Cell Tumor Burden and Variant Allele Frequency (VAF) at Diagnosis and Relapse

Evaluating the tumor burden constitutes an important task, particularly in cHL in which HRS cells represent a minority within the prominent tumor microenvironment (TME). Following previous methods published by our group [31,32], we quantified the HRS percentage by using whole-slide imaging (WSI) and digital image analysis. The median tumor burden (HRS cell percentage) for all the cases was 4.3 (95% CI: 5.3–21.8), without adjusting to a normal distribution (Kolmogorov–Smirnov, *p* = 0.000). Cases showing chemoresistance presented with a higher tumor burden (median tumor burden: 5.60, 95% CI: 5.18–25.02) when compared with cHL patients in complete remission (median: 3.62, 95% CI: −5.70–27.74), but the differences were not statistically significant (*p* = 1.0).

Interestingly, in EBV-negative cHL, the HRS cell percentage was lower in EBV-positive cases (median tumor burden, EBV-negative cHL: 4.83, 95% CI: 1.33–21.05; EBV-positive cHL: 2.83, 95% CI: −1.25–28.28), but the differences did not reach statistical significance (*p* = 0.4) probably because of an effect of the small sample size. Neither the variant allele frequencies (VAFs) of the pathogenic variants identified (*p* = 0.1) nor the tumoral burden at diagnosis and relapse (*p* = 0.8) were statistically significantly associated in the cases evaluated.

### 2.6. Temporal Dynamics and Clonal Selection in Relapsed/Refractory Classic Hodgkin Lymphoma

In order to evaluate the evolution of mutated clones in chemorefractory cHL, we examined paired samples obtained at diagnosis and relapse. First, we found a reduction in the number of somatic mutations identified at relapse when compared to the onset of the disease. A total of 81 variants were identified at diagnosis, while 51 variants were identified at relapse, suggesting that selection of mutated clones during the evolution of the disease is a common event in chemorefractory cHL. We also revised the pathogenic variants at diagnosis and relapse for each case. From the 47 genes of the targeted NGS panel, just 1 of them (SF3B1) appeared both at diagnosis and relapse, and the variant allele frequency at relapse was <1%.

Longitudinal assessment of individual cases allowed for differentiating distinct evolutionary patterns. On the one hand, in certain cases (N3–N6), clones frequently appeared harboring mutations at relapse that were not present at diagnosis (Figure S5). On the other hand, clonal selection and expansion of subclones previously not identified at diagnosis were the most remarkable events (N1, N2, N7–N10) (Figure S6). Table 2 shows the mutations identified at diagnosis and paired relapse tissue samples in refractory/relapsed cHL patients.

## 3. Discussion

The identification of the factors that determine poor therapy responses in cHL is still a major research goal. Despite the history of cHL [33] constitutes a paradigm that cancer is a curable entity, 10–20% of patients experience recurrences during follow-up and deaths are still observed [3,4].

A growing body of knowledge has been accumulating during the last ten years on the molecular features of cHL [34,35,36,37,38]. However, the translation of these advances to the clinical setting has not yet been realized, and today, the current risk stratification models [39,40,41] and clinical management are based on clinical, analytical and imaging data.

Affordability and progressive adoption of sequencing technologies in health systems [42], mainly through targeted NGS sequencing panels specifically designed for B-cell lymphomas [9,10], are likely to change this scenario in the coming years. With this aim, we examined the genomic profile of cHL and compared data obtained among patients achieving long-term remission and relapsed/refractory cases. Furthermore, since dynamic evolution and selection of resistant clones after therapy pressure are well-known events in cancer progression [43,44], we also longitudinally sequenced paired diagnosis–relapse tissue samples to examine evolutionary patterns in chemoresistant cHL patients.

A significant proportion of the cases analyzed showed abnormalities in genes linked to the NOTCH pathway. This finding has been recurrently reported in cHL [13,14,15,16]. NOTCH constitutes an ancient and highly developmentally conserved pathway [45,46], firstly linked to human cancer in T acute lymphoblastic leukemia (T-ALL) [47,48]. It regulates the cell cycle, initiates tumor progression and facilitates tumoral angiogenesis and tumor invasion [49]. In cHL, NOTCH1 expression in HRS cells promotes cell proliferation by interacting with its ligand JAGGED1 [12]. Interestingly, JAGGED1 has been demonstrated to be expressed both by HRS cells themselves and cells resident in the tumoral microenvironment, such as epithelioid cells neighboring HRS cells [50].

In accordance with the NOTCH signature of HRS cells, Spina et al. [6] found mutations in the NOTCH pathway in 20.0% of newly diagnosed cHL patients, whereas a higher proportion (50.0%) of the patients from our cohort were identified to harbor mutations in NOTCH genes. Meanwhile, in another study by Alcoceba et al. [34], NOTCH1 and NOTCH2 genes were not considered within the genes included in the targeted sequencing panel, limiting the comparison of detection rates. Our data extend and reinforce the notion that NOTCH is a major and recurrently mutated pathway in cHL, and they support the development of therapeutic agents targeting NOTCH members.

In relapsed/refractory cHL tumor samples, CREBBP was the most frequently mutated gene, being affected in 60.0% of the patients. Remarkably, in this subgroup of patients, the SF3B1, ARID1A and EP300 genes, all of them associated with epigenetic modifications of gene expression, were also found to be mutated in a significant proportion of the cases. Our data are in accordance with recent studies that highlight the central role of epigenetic changes both in the pathobiology of cHL [35] and, especially, in the acquisition of chemoresistance. Epigenetic modifications (i.e., DNA methylation) seem to be essential for HRS survival and phenotype acquisition [21,22]. In particular, CREBBP regulates gene expression by chromatin remodeling exerted via histone acetyltransferase (HAT) enzymes [51]. Furthermore, loss-of-function mutations in CREBBP lead to increased development of B-cell lymphomas in murine models [52]. Our data are in accordance with previous results presented by Mata et al. [8]. The authors identified mutations of CREBBP and EP300 as the most frequent in relapsed/refractory cHL patients (30.0% and 40.0%, respectively). These results are unsurprising, as cHL constitutes a germinal center B-cell lymphoma, and CREBBP has been evidenced to modulate cell fate decisions in GC lymphomagenesis [52]. Collectively, these results provide a rational basis for current clinical trials (NCT05355051, NCT02961101 and NCT03250962) that evaluate the combination of DNA methylation and histonedeacetyltransferase (HDAC) inhibitors with anti-PD1 monoclonal antibodies (mABs) in the setting of relapsed/refractory cHL.

Nonetheless, several limitations to the extant research must be addressed in future studies. First, this study was limited by the small sample size. We did not find differences in survival distributions depending on the mutational status of CREBBP, which could have been related to a low statistical power as an effect of the sample size. Furthermore, in the most frequent mutated genes in relapsed/refractory cHL patients (CREBBP (60%), NOTCH2 (50%), PTPRD (50%), SF3B1 (50%), and ARID1A, EP300, NOTCH1, PLCG2 (30% each one)), no statistically significant differences were observed in our cohort. Second, a recent study [53] reported that a significant proportion of recurrences in cHL were secondary primary cHL, clonally unrelated to the primary tumor. Using the EuroClonality IG–next-generation sequencing assay, the authors demonstrated that a clonal relationship could not be established in ~60% of cHL recurrences after two years. In our study, in 6 of the 10 patients (60%), mutations at diagnosis were also identified at relapse (patients 1, 2 and 7–10). These mutations involved the NOTCH1, NOTCH2, CREBBP, XPO1 and PTPRD genes. However, clonality assessment of cHL recurrences constituted a limitation of our work because IGH-clonality assessment was not considered in the design of the NGS panel evaluated. Consequently, the distinction between primary unrelated secondary cHL and bona fide cHL recurrences that were clonally related could not be formally derived from our NGS approach.

Broader approaches to sequencing, such as whole-exome sequencing (WES) and whole-genome sequencing (WGS), allow for the discovery of previously undescribed pathogenic variants and could provide important and complementary information that will be useful for developing targeted sequencing. The fact to employ a targeted approach compromises this possibility and, thus, needs to be complemented with WGS and WES approaches to comprehensively evaluate the mutational landscape of cHL. Lastly, circulating tumor DNA (ctDNA) strategies are emerging as a noninvasive and feasible approach for cHL genotyping and monitoring of the treatment response. In a recent investigation by Alig et al. [54], when using ctDNA sequencing, two distinct cHL subtypes (H1 and H2) were distinguished and correlated with pathological, clinical and prognostic features. Interestingly, HL2 patients presented inferior outcomes and were enriched in mutations involving TP53 and epigenetic regulators such as KMT2D. The application of the same NGS panel employed in this project to liquid biopsies of cHL patients with the objective of monitoring the treatment response and achieving early identification of molecular biomarkers constitutes an ongoing avenue of research in our department.

## 4. Materials and Methods

### 4.1. Patients and Samples

This study had a retrospective observational design. Samples were selected to compare the mutational profile of relapsed/refractory cHL and cHL patients achieving a complete metabolic response after induction treatment. The availability of biological material (formalin-fixed paraffin-embedded lymph node samples) and clinical data in the electronic health record (EHR), age > 18 years old and first-line chemo-radiotherapy with curative intention were the study inclusion criteria. A total of 32 samples (HL1–HL32) from 22 patients diagnosed with cHL (N1–N22) were analyzed. In relapsed/refractory cHL, paired samples from diagnosis and recurrence of the disease were evaluated. The diagnoses were made in accordance with the 5th edition of the WHO Classification of Haematolymphoid Tumours (WHO-HAEM5) [19].

The following clinical and histopathological variables were studied from each case: sex, age, histological subtype, EBV infection of HRS cells, as determined by the immunohistochemical expression of EBV LMP-1, Ann Arbor stage with Cotswolds modifications, presence of B symptoms at diagnosis (fever, drenching night sweats or loss of more than 10% of body weight over 6 months prior to diagnosis), bulky disease (mass in the chest that is one-third the width of the chest, or any lymph node mass greater than 10 cm) and therapy response after first-line treatment.

All the patients included in this study were treated with an adriamycin, bleomycin, vinblastine and dacarbazine (ABVD) regimen as induction therapy. All the samples and data were obtained following the technical and ethical procedures of the local institutions and in accordance with the Declaration of Helsinki. This study was approved by the Cadiz Research Ethics Committee (protocol code 1167-N-21).

### 4.2. DNA Extraction and Construction of Libraries

Tissue sections were deparaffinized with xylene and alcohol, while proteins were digested with 4 mL of protease per specimen and centrifuged for 30 s at 10,000× *g*. DNA and RNA were extracted using the MagMAXt FFPE DNA/RNA Ultra Kit (Thermo Fisher Scientific, Austin, TX, USA) following the manufacturer’s protocol. Fluorometric quantitation was performed using a Qubit 2.0 Fluorometer with Qubit dsDNA/RNA HS Assay Kits and the Genejet RNA Cleanup and Concentration Micro Kit (Thermo Fisher Scientific, Inc.) according to the manufacturer’s protocols, and we considered it appropriate when the nucleic acid concentration was >10 ng/μL^−1^.

### 4.3. Library Preparation and Templating

DNA and RNA libraries were constructed using the Oncomine Focus Library Assay Chef Ready and Ion 510 & Ion 520 & Ion 530 Kit-Chef (Thermo Fisher Scientific, Austin, TX, USA) according to the manufacturer’s instructions. At least 10 ng of genomic DNA and RNA was used per microplate. Unique Ion Code Barcode 1–8 (DNA) and Ion Code Barcode 9–16 (RNA) were used to be ligated to the amplicons and subsequently purified and equalized to ~100 pM. All steps in library and template preparation were automated using Ion Chef Robot (Thermo Fisher Scientific, Austin, TX, USA).

### 4.4. Next-Generation Sequencing, Variant Calling and Variant Categorization

A total of eight DNA and nine RNA uniquely barcoded library samples were pooled for sequencing per run on an Ion 530 chip (Thermo Fisher Scientific, Austin, TX, USA). After chip loading, sequence analysis was performed using the Ion GeneStudio S5 System (Thermo Fisher Scientific, Austin, TX, USA). In this study, NGS studies were performed in bulk tissue samples. When analyzing cHL samples, we previously cut and observed anti-CD30 tissue-stained specimens in order to select areas enriched in HRS cells within the histopathological slides. Once observed in digital scanners (PANNORAMIC ^®^ 250 Flash III DX scanner, 3D Histech Ltd., Budapest, Hungary), we selected these areas and performed sequencing only in HRS-enriched regions of the formalin-fixed paraffin-embedded (FFPE) specimens. Prior to sequencing, we ensured that each case had at least 10 ng of genomic DNA belonging to a monoclonal population. However, it is not possible to reject the possibility that mutations in subclones may not have been identified. A targeted sequencing panel including 47 genes recurrently mutated in B-cell neoplasms was specifically designed for this project (Table S2). Genes were selected based on the previous literature and a systematic review of documents that reported the results of NGS studies in cHL [11]. The cHL targeted panel was designed to cover coding exons and splice sites of 47 genes clustered in 9 functional categories, related to recurrently altered pathways in the pathogenesis of cHL: cell cycle control (CCND1, CDKN2A, TP53), apoptosis (ABL1, BIRC3, CASP8, BCL2, BCL10, FAS), immune system control (CD79A, CD79B, CSF1R, MYD88, B2M, TCF3, CD19, CD38), the BTK and NFKB signaling pathways (BTK, CARD11, CYLD, IL32, LCP1, NFKBIE, NFKBIA, TNFAIP3, TRAF2), the JAK-STAT pathway (CSF2, CSF2RB, STAT6, PTPRD), transcription factors (FOXO1, KLF2, MEF2B, MYB, MYC), epigenetics (CREBBP, EP300, ARID1A, EZH2), the NOTCH signaling pathway and MAP kinases (NOTCH1, NOTCH2, BRAF, CXCR4, ID3) and nuclear RNA, splicing and signaling molecules (XPO1, SF3B1, PLCG2). Sequencing Binary Alignment Map (BAM) files were analyzed with the Ion Report Software (Version 5.16, Thermo Fisher Scientific, Austin, TX, USA) and uploaded to the Ion Reporter Server cloud system under our institutional license (https://ionreporter.thermofisher.com (accesed on 17 February 2024)). We used the Oncomine Focus–520–w3.1–DNA and Fusions–Single Sample workflow, and analysis was performed using Oncomine Variant Annotator v3.1 in Ion Reporter. For a detailed identification and manual review of variants, the “No filter” option was selected as the filter chain, and tab-separated values (.TSV) files, including all variants, were downloaded from the Ion Reporter System and re-analyzed using a robust bioinformatics pipeline, to discard synonymous and intronic variants and only select genomic variants located in coding exon regions (Supplementary Methods S1). Prediction of the pathogenicity of point mutations we discovered was performed by using SIFT [55], Polyphen-2 [56] and Grantham [57]. To exclude polymorphic variants, dbSNP [58] was employed.

### 4.5. Immunohistochemistry

Immunohistochemical analysis was performed using heat-induced epitope retrieval following standard procedures, employing antiCD30 (Ber-H2) mouse monoclonal primary antibody (G13408Z, Ventana Medical Systems Inc., Tucson, AZ, USA). The evaluation of EBV presence was performed using anti-human mouse Epstein–Barr virus/LMP1 monoclonal antibody (Clone CS1-4) (Master Diagnostica, Inc., Granada, Spain).

The detection of protein expression was performed with the automated immunostainer BenchMark ULTRA IHC/ISH System (Ventana Medical Systems Inc., Tucson, AZ, USA), following the manufacturer’s protocols.

### 4.6. Endpoints

The progression-free survival (PFS) was used as a clinical endpoint for survival analysis. The PFS was defined as the time interval between the initial histopathological diagnosis and the first progression or relapse after achieving a complete metabolic response. The evaluation of the response to the treatment was performed by 18F-Fluorodeoxyglucose (18F-FDG) positron emission tomography/computed tomography (PET/CT) following the revised Cheson criteria [59].

### 4.7. Statistical Analysis

Descriptive statistics were generated and data were reported as percentages (qualitative variables) and the mean, standard deviation (SD) and range (quantitative variables). For statistical inference, the χ^2^ test and the Fisher exact test were used for the comparison between categorical variables. Normality was assessed by the Kolmogorov–Smirnov test. For the comparison of quantitative variables, the Spearman correlation test was applied. In order to analyze and compare survival distributions according to the sequencing data, the Kaplan–Meier method with the log-rank test was employed. All p-values were two-sided, and a level of probability below 0.05 was considered significant. The IBM SPSS software (version 15.0) (SPSS Inc., Chicago, IL, USA) and R package (version 4.1.3) were used to perform the statistical analysis.

## 5. Conclusions

The identification of the genomic background of relapsed/refractory cHL still constitutes a major research goal, as 10–20% of patients experience recurrences with current multiagent protocols. In this study, we expand and reinforce the notion that NOTCH is a hallmark and frequently mutated pathway in cHL. Furthermore, we identify frequent pathogenic variants involving CREBBP and EP300, both of them associated with epigenetic abnormalities in GC-derived B-cell lymphomas such as cHL. Lastly, in cHL cases showing chemoresistance to frontline chemotherapy, we demonstrate that clonal selection and the appearance of novel mutant clones previously not identified at diagnosis is a common event during the course of the disease. The results of this study expand the current biological knowledge of cHL and support the development of personalized therapies targeting epigenetic mediators.

## Figures and Tables

**Table 1:** Characteristics of the patients included in this study.

Characteristic	Result
Female sex, no. (%)	13 (59.1)
Age, years, median (range)	33 (19–69)
Ann Arbor stage, no. (%)	
Initial stages (I–II)	10 (45.5)
Advanced stages (III–IV)	12 (54.5)
Histology, no. (%)	
Nodular sclerosis	17 (77.3)
Lymphocyte-rich	2 (9.1)
Not otherwise specified (NOS)	3 (13.6)
Bulky disease, no. (%)	19 (86.4)
B symptoms, no. (%)	14 (63.6)
Epstein–Barr virus latent membrane protein-1, no. (%)	6 (27.3)
Treatment response, no. (%)	
Complete metabolic response	12 (54.5)
Relapse/refractory	10 (45.5)

No., number.

**Table 2:** Mutations identified in paired diagnosis–relapse tissue samples of refractory/relapsed classic Hodgkin lymphoma (cHL). Diagnosis samples correspond from N1 to N10 and relapse samples correspond from N11 to N20. * Corresponds to a gene mutated at diagnosis and relapse.

Refractory Cases	Genes Mutated at Diagnosis (N1–N10)	Genes Mutated at Relapse (N11–N20)
Patient 1 (Samples N1 and N11)	NOTCH2 * (W2436Ter) CREBBP * (P1943S)	NOTCH2 * (C745Y) CREBBP * (S2338F)
Patient 2 (Samples N2 and N12)	NOTCH1 (S2341F) ARID1A (S740F) CREBBP (S2361F) EZH2 (R690C) NOTCH2 *(F1017S) SF3B1 (S541F) CSF1R (C278Y) MYB (S245F) TNFAIP3 (C627Y) PTPRD (S874F) CSF2RB (S322F) EP300 (C1163Y)	NOTCH2 * (S2437L)
Patient 3 (Samples N3 and N13)	NOTCH1 (C1363Y) PLCG2 (Y293C) EP300 (S1642F)	PTPRD (G61R)
Patient 4 (Samples N4 and N14)	NOTCH2 (S2437L) ARID1A (G151R) KLF2 (R145C)	NOTCH1 (C398Y)
Patient 5 (Samples N5 and N15)	CREBBP (S1917L) SF3B1 (S57L)	ARID1A (S1570F) TNFAIP3 (C612Y) EP300 (C1177Y)
Patient 6 (Samples N6 and N16)	PTPRD (M282I) CYLD (S544L) EP300 (L1788F) BTK (E488K)	NOTCH1 (C1521Y)
Patient 7 (Samples N7 and N17)	NOTCH2 * (S2437L) CREBBP (A884T) ARID1A (G151E) CARD11 (D446N)	NOTCH2 * (R568C) BTK (S371F) SF3B1 (R568C) PTPRD (S623F) BCL10 (R25C)
Patient 8 (Samples N8 and N18)	SF3B1 (C1123Y) PTPRD (R1181C) XPO1 * (R553C) CD38 (C99Y) EZH2 (S114F) CYLD (S384F) PLCG2 (S152F) CSF2RB (C289Y)	XPO1 *(P552L) CREBBP (P1476L)
Patient 9 (Samples N9 and N19)	NOTCH1 * (C440Y) NOTCH2 (S2078F) PTPRD (C1465Y) XPO1 (C595Y) CXCR4 (S330F) SF3B1 (S637F) TNFAIP3 (R562C) BRAF (C748Y) CASP8 (C371Y) CARD11 (R35C) FOXO1 (S363F) CREBBP (C398Y) PLCG2 (S1179F) TCF3 (S157F) BTK (R641C)	NOTCH1 * (S2530F)
Patient 10 (Samples N10 and N20)	SF3B1 (R568C) PTPRD * (R174C) CREBBP * (S566F)	NOTCH1 (S1856F) NOTCH2 (S2421F) SF3B1 (S611F) CXCR4 (S319F) CSF1R (S840F) ABL1 (S438F) PTPRD * (R1181C) CREBBP * (S1432F) EP300 (C1177Y) ARID1A (S1604F) BRAF (S125F) PLCG2 (S1179F)

## Data Availability

The data presented in this study are available on request from the corresponding author.

## Supplementary Materials

The following supporting information can be downloaded at: https://www.mdpi.com/article/10.3390/ijms25052457/s1.
