# Supplementary material for: Pathogenic Variants Associated with Epigenetic Control and the NOTCH Pathway Are Frequent in Classic Hodgkin Lymphoma
Source: Int J Mol Sci. 2024 Feb 20;25(5):2457. doi: 10.3390/ijms25052457 (PMC10931057; doi:10.3390/ijms25052457)

Supplementary Material

Pathogenic Variants Associated with Epigenetic Control and the NOTCH Pathway Are Frequent in Classic Hodgkin Lymphoma

Antonio Santisteban-Espejo 1,2,3,*, Irene Bernal-Florindo 3,4, Pedro Montero-Pavon 4 , Jose Perez-Requena 1, Lidia Atienza-Cuevas 1, Maria del Carmen Fernandez-Valle 5, Ana Villalba-Fernandez 1 and Marcial Garcia-Rojo 3,4

1 Department of Pathology, Puerta del Mar University Hospital, 11009 Cadiz, Spain; jose.perez.sspa@juntadeandalucia.es (J.P.-R.); anavillalbafernandez@hotmail.com (A.V.-F.)
2 Department of Medicine and Surgery, Faculty of Medicine, University of Cadiz, 11003 Cadiz, Spain
3 Institute of Research and Innovation in Biomedical Sciences of the Province of Cadiz (INiBICA), 11009 Cadiz, Spain; iriberni@hotmail.com (I.B.-F.); marcial.garcia.sspa@juntadeandalucia.es (M.G.-R.)
4 Department of Pathology, Jerez de la Frontera University Hospital, 11407 Cadiz, Spain; pmonteropavon@gmail.com
5 Department of Hematology and Hemotherapy, Puerta del Mar University Hospital, 11009 Cadiz, Spain; macafeva@hotmail.com

Correspondence: antonio.santisteban@uca.es

Supplementary Methods 1. Bioinformatics analysis, variant calling and variant categorization

Sequencing Binary Alignment Map (BAM) files were analyzed with the Ion Report Software (Version 5.16, Thermo Fisher Scientific, Texas, USA). For variant calling, Oncomine Variant Annotator v3.1 was used. In order to identify and annotate the SNVs, indels and CNVs, the following filter chains were applied in Ion Reporter: frequency cutoff for supporting a variant, SNV 0.04, indel 0.07, hotspot 0.03; total coverage required of reads or no-call, SNV 15, indel 15, hotspot 15; proportion of variant alleles coming overwhelmingly from one strand, SNV 0.96, indel 0.9, hotspot 0.96 for SNV and indel calls; and median of the absolute values of all pairwise differences < 0.4; 5% confidence interval CNV ploidy ≥ gain of 2 over normal for CNV calling.

We restricted the analyses to variants with a total coverage of at least 100x. Only genomic variants in coding exon regions were considered. Intronic variants were discarded and variants with an allele frequency < 1% or > 40% were not considered. Because of they constitute single nucleotide polymorphisms (SNPs) or somatic variants at very low frequency (AF<1%) in cells other than the tumoral fraction of cHL, or also represent germline mutations (AF> 40%) these variants were not considered in the analysis, as probably not informing about the tumoral clone of the cHL tissue samples evaluated. Additionally, for exclusion of SNPs, information available in dbSNP was also consulted. Then, variants obtained were filtered according to the functional consequence at the aminoacid sequence, and only nonsynonymous variants were considered for further evaluation. Using this pipeline, from an initial set of 38384 variants, we finally obtained 226 variants (58.9%) corresponding to the 32 cHL samples analyzed (HL1-HL32). This stringent filtering process, the concordance of variants obtained in paired samples and the identification of recurrent mutations previously described in cHL, confirm the robustness of the analysis, even considering the possibility of losing variants not considered that could contribute to intratumoral heterogeneity or explain, in part, disease progression.

Supplementary legends

1. Supplementary Table 1 (Table S1). Targeted next-generation sequencing panel for B-cell neoplasms developed for the project.

2. Supplementary Figure 1 (Figure S1). Variant allele frequency of classic Hodgkin lymphoma cases analyzed (n=32).

3. Supplementary Figure 2 (Figure S2). Coverage of classic Hodgkin lymphoma cases analyzed (n=32).

4. Supplementary Figure 3 (Figure S3). Percentage of mutated cases of classic Hodgkin lymphoma (n=32).

5. Supplementary Figure 4 (Figure S4). Co-occurrence matrix. The probability of co-occurrence between the mutations of the genes defined in the targeted sequencing panel is shown in red color.

6. Supplementary Table 2 (Table S2). Description of the variants identified in classic Hodgkin lymphoma (n=32).

7. Supplementary Figure 5 (Figure S5). Clonal evolution of chemorefractory cHL. Cases with novel mutations at relapse previously not identified at diagnosis. Time evolution from diagnosis to relapse is shown in the x-axis. The y-axis shows the mean allele frequency of the variants identified. Common ancestral clone is shown in black color. Mutations identified at diagnosis and relapse are shown in blue color and red color, respectively.

8. Supplementary Figure 6 (Figure S6). Clonal evolution of chemorefractory cHL. Cases with selection of mutations at relapse previously identified at diagnosis (clonal selection). Time evolution from diagnosis to relapse is shown in the x-axis. The y-axis shows the mean allele frequency of the variants identified. Common ancestral clone is shown in black color. Mutations identified at diagnosis and relapse are shown in blue color and red color, respectively.

1. Supplementary Table 1 (Table S1).

Gene	Chromosome	Number of amplicons	Total bases	Covered bases	Missed bases	Coverage
ID3	Chr1	5	400	400	0	1.000
ARID1A	Chr1	87	7258	7187	71	0.969
BCL10	Chr1	10	762	762	0	1.000
NOTCH2	Chr1	102	8149	7958	191	0.731
XPO1	Chr2	51	3696	3674	22	0.916
CXCR4	Chr2	13	1126	1126	0	1.000
SF3B1	Chr2	68	4505	4478	27	0.961
CASP8	Chr2	24	1913	1889	24	0.984
MYD88	Chr3	14	1054	1054	0	1.000
CD38	Chr4	15	1063	1063	0	1.000
CSF2	Chr5	9	515	515	0	1.000
CSF1R	Chr5	51	3339	3339	0	1.000
NFKBIE	Chr6	21	1623	1623	0	1.000
MYB	Chr6	38	2606	2596	10	0.984
TNFAIP3	Chr6	26	2533	2515	18	0.990
CARD11	Chr7	54	3945	3945	0	1.000
BRAF	Chr7	42	2661	2655	6	0.997
EZH2	Chr7	40	2636	2636	0	1.000

MYC	Chr8	15	1425	1425	0	1.000
PTPRD	Chr9	84	6539	6516	23	0.996
CDKN2A	Chr9	11	1012	1008	4	0.996
ABL1	Chr9	45	3769	3759	10	0.991
NOTCH1	Chr9	111	8348	8274	74	0.973
TRAF2	Chr9	21	1706	1706	0	1.000
FAS	Chr10	17	1188	1188	0	1.000
CCND1	Chr11	14	988	988	0	1.000
BIRC3	Chr11	28	1975	1971	4	0.997
STAT6	Chr12	42	2964	2955	9	0.996
FOXO1	Chr13	19	2608	2529	79	0.939
LCP1	Chr13	30	2184	2184	0	1.000
NFKBIA	Chr14	16	1074	1074	0	1.000
B2M	Chr15	7	420	420	0	1.000
IL32	Chr16	9	805	605	200	0.896
CREBBP	Chr16	95	7949	7926	23	0.995
CD19	Chr16	32	1954	1954	0	1.000
CYLD	Chr16	43	3211	3144	67	0.978
PLCG2	Chr16	62	4438	4438	0	1.000
TP53	Chr17	24	1503	1503	0	1.000
CD79B	Chr17	12	813	813	0	1.000
BCL2	Chr18	9	793	793	0	1.000
TCF3	Chr19	35	2572	2567	5	0.997
KLF2	Chr19	12	1128	968	160	0.936
MEF2B	Chr19	16	1267	1267	0	1.000
CD79A	Chr19	12	781	781	0	1.000
CSF2RB	Chr22	38	2954	2954	0	1.000
EP300	Chr22	97	7865	7865	0	1.000
BTK	ChrX	33	2462	2462	0	1.000

2. **Supplementary Figure 1 (Figure S1).**

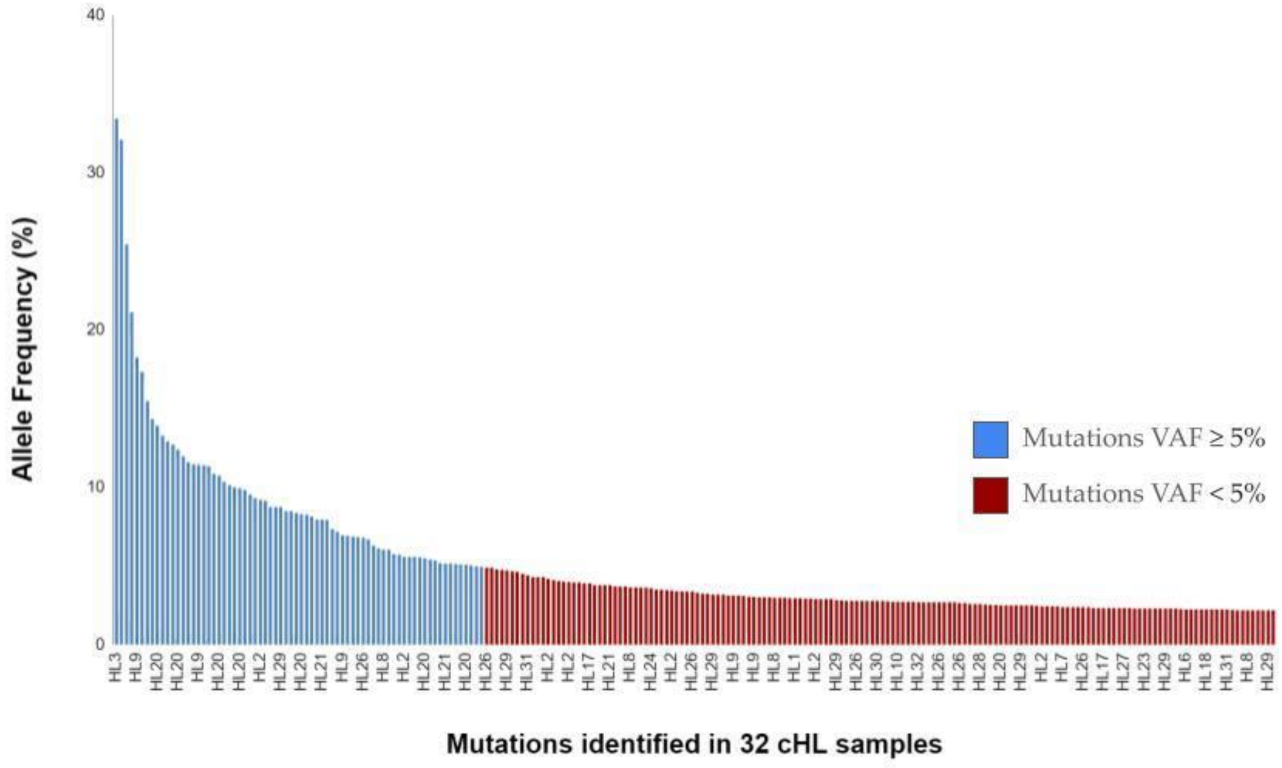

3. **Supplementary Figure 2 (Figure S2).**

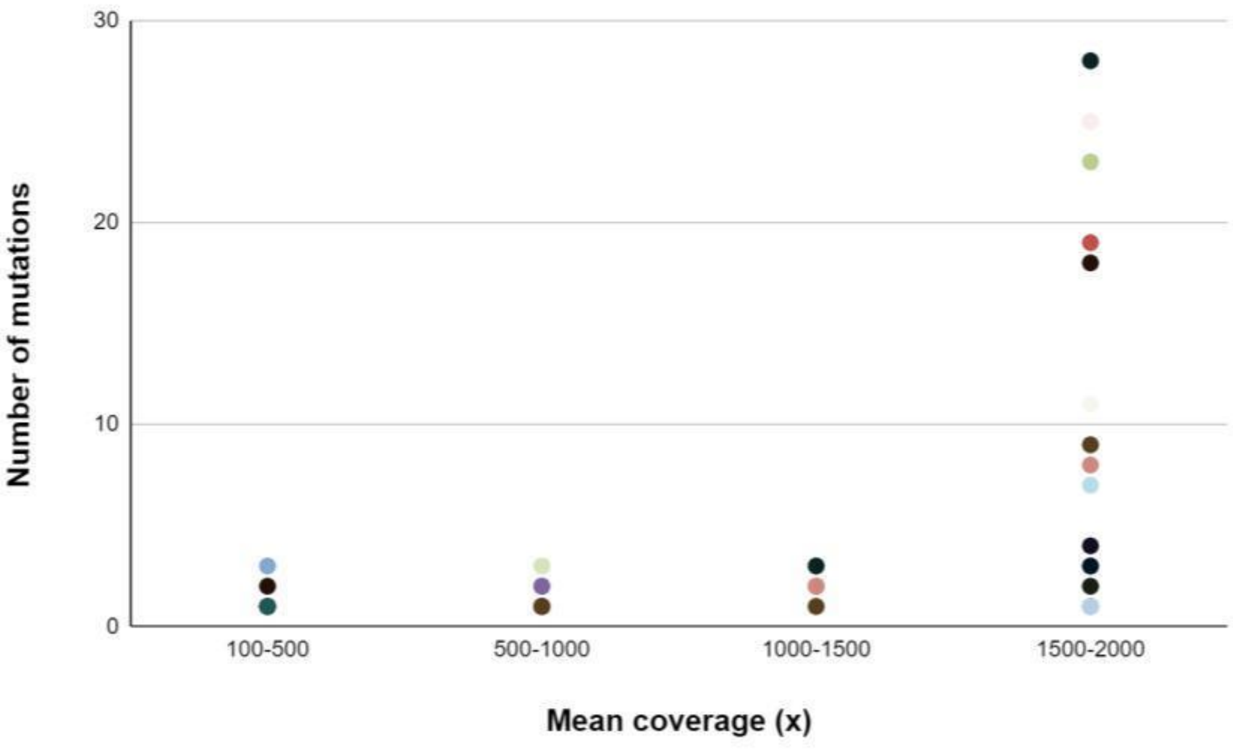

4. Supplementary Figure 3 (Figure S3).

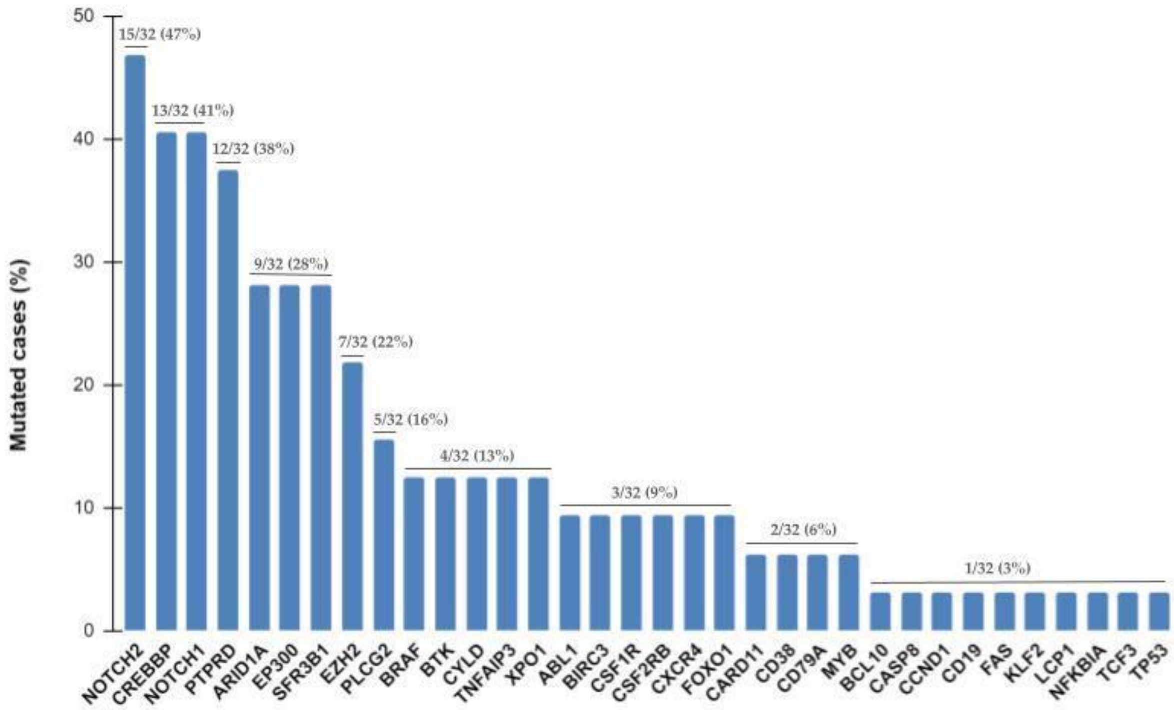

5. Supplementary Figure 4 (Figure S4).

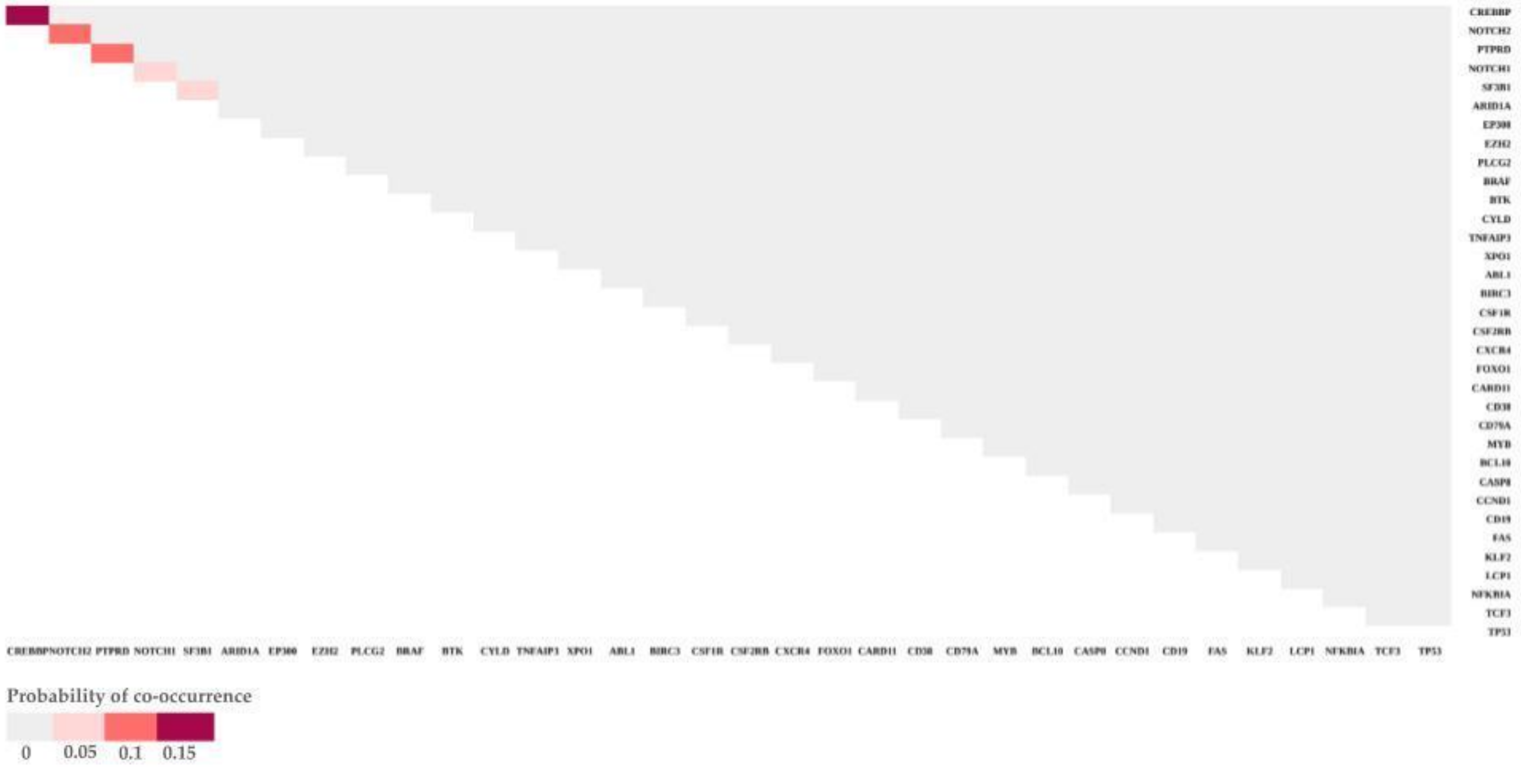

6. Supplementary Table 2 (Table S2).

Case	Gen	Locus	Change	Allele frequency	Coverage	Type	Aminoacid change	Variant effect
HL1	NOTCH2	chr1:120458038	C>T	2.96	608	SNV	W2436Ter	unknown
HL1	NOTCH2	chr1:120458040	G>A	2.96	608	SNV	D2435=	unknown
HL1	CREBBP	chr16:3779221	G>A	7.96	113	SNV	P1943S	missense
HL2	ARID1A	chr1:27087932	C>T	2.55	2000	SNV	S740F	missense
HL2	ARID1A	chr1:27101475	C>T	3.15	2000	SNV	S1586F	missense
HL2	ARID1A	chr1:27101517	C>T	5.00	2000	SNV	S1600F	missense
HL2	NOTCH2	chr1:120483311	A>G	2.78	1976	SNV	F1017S	unknown
HL2	SF3B1	chr2:198268406	G>A	3.45	2000	SNV	S541F	missense
HL2	CSF1R	chr5:149456895	C>T	2.45	2000	SNV	C278Y	missense
HL2	MYB	chr6:135513668	C>T	4.97	1770	SNV	S245F	missense
HL2	TNFAIP3	chr6:138200462	G>A	2.70	2000	SNV	C627Y	missense
HL2	EZH2	chr7:148506444	G>A	6.88	1279	SNV	R690C	missense
HL2	EZH2	chr7:148511175	C>T	5.60	1999	SNV	C576Y	missense
HL2	PTPRD	chr9:8486196	G>A	9.21	1716	SNV	S874F	missense
HL2	NOTCH1	chr9:139391169	G>A	4.20	2000	SNV	S2341F	missense
HL2	NOTCH1	chr9:139396781	G>A	5.74	122	SNV	S1776F	missense
HL2	NOTCH1	chr9:139399987	C>T	2.90	2000	SNV	C1454Y	missense
HL2	NOTCH1	chr9:139400200	C>T	5.75	1253	SNV	C1383Y	missense
HL2	NOTCH1	chr9:139401046	C>T	4.05	2000	SNV	C1316Y	missense
HL2	NOTCH1	chr9:139412702	C>T	2.60	1999	SNV	C381Y	missense
HL2	NOTCH1	chr9:139418369	C>T	4.30	1999	SNV	C68Y	missense
HL2	CREBBP	chr16:3777966	G>A	4.00	2000	SNV	S2361F	missense
HL2	CREBBP	chr16:3778035	G>A	3.90	2000	SNV	S2338F	missense
HL2	CREBBP	chr16:3779601	C>T	3.05	2000	SNV	C1816Y	missense
HL2	CSF2RB	chr22:37326825	C>T	3.40	2000	SNV	S322F	missense
HL2	EP300	chr22:41553399	G>A	7.35	952	SNV	C1163Y	missense
HL3	NOTCH1	chr9:139400260	C>T	32.11	1118	SNV	C1363Y	missense
HL3	PLCG2	chr16:81925087	A>G	9.56	952	SNV	Y293C	missense
HL3	EP300	chr22:41572396	C>T	33.45	1115	SNV	S1642F	missense
HL4	ARID1A	chr1:27023345	G>A	2.99	234	SNV	G151R	missense
HL4	NOTCH2	chr1:120458035	GA>AA	6.94	577	SNV	S2437L	unknown
HL4	KLF2	chr19:16436384	C>T	4.03	571	SNV	R145C	missense
HL5	SF3B1	chr2:198288557	G>A	2.20	2000	SNV	S57L	missense
HL5	CREBBP	chr16:3779298	G>A	2.29	1744	SNV	S1917L	missense
HL6	PTPRD	chr9:8521392	C>T	2.83	1271	SNV	M282I	missense
HL6	CYLD	chr16:50815278	C>T	9.32	472	SNV	S544L	missense
HL6	EP300	chr22:41573077	C>T	2.20	2000	SNV	L1788F	missense
HL6	BTK	chrX:100611144	C>T	2.25	2000	SNV	E488K	missense
HL7	ARID1A	chr1:27023346	G>A	5.12	254	SNV	G151E	missense

HL7	NOTCH2	chr1:120458035	G>A	5.58	448	SNV	S2437L	unknown
HL7	CARD11	chr7:2976676	C>T	2.40	1794	SNV	D446N	missense
HL7	CREBBP	chr16:3820801	C>T	5.42	369	SNV	A884T	missense
HL8	XPO1	chr2:61719526	G>A	6.06	1122	SNV	R553C	missense
HL8	SF3B1	chr2:198260951	C>T	4.30	2000	SNV	C1123Y	missense
HL8	SF3B1	chr2:198285835	G>A	3.00	1999	SNV	S73F	missense
HL8	CD38	chr4:15818196	G>A	2.35	1999	SNV	C99Y	missense
HL8	EZH2	chr7:148529748	G>A	3.65	1287	SNV	S114F	missense
HL8	PTPRD	chr9:8465639	G>A	2.20	2000	SNV	R1181C	missense
HL8	PTPRD	chr9:8486196	G>A	2.25	2000	SNV	S874F	missense
HL8	CYLD	chr16:50813597	C>T	2.80	1998	SNV	S384F	missense
HL8	PLCG2	chr16:81892744	C>T	2.90	1553	SNV	S152F	missense
HL8	CSF2RB	chr22:37326726	G>A	2.75	2000	SNV	C289Y	missense
HL9	NOTCH2	chr1:120459112	G>A	2.25	2000	SNV	S2078F	unknown
HL9	NOTCH2	chr1:120508117	C>T	11.60	2000	SNV	C547Y	unknown,
HL9	NOTCH2	chr1:120512166	G>A	8.50	2000	SNV	S359F	unknown
HL9	XPO1	chr2:61719273	C>T	2.70	1999	SNV	C595Y	missense
HL9	XPO1	chr2:61720185	G>A	4.90	2000	SNV	R417C	missense
HL9	CXCR4	chr2:136872509	G>A	12.93	1083	SNV	S330F	missense
HL9	CXCR4	chr2:136872542	G>A	4.30	2000	SNV	S319F	missense
HL9	SF3B1	chr2:198267447	G>A	7.20	2000	SNV	S637F	missense
HL9	SF3B1	chr2:198267525	G>A	3.40	2000	SNV	S611F	missense
HL9	CASP8	chr2:202149671	G>A	6.95	2000	SNV	C371Y	missense
HL9	TNFAIP3	chr6:138200266	C>T	10.87	1996	SNV	R562C	missense
HL9	TNFAIP3	chr6:138200417	G>A	6.85	1999	SNV	C612Y	missense
HL9	CARD11	chr7:2987326	G>A	6.05	2000	SNV	R35C	missense
HL9	BRAF	chr7:140434455	C>T	4.50	2000	SNV	C748Y	missense
HL9	BRAF	chr7:140534539	G>A	7.93	1993	SNV	S125F	missense
HL9	PTPRD	chr9:8376719	C>T	3.05	2000	SNV	C1465Y	missense
HL9	PTPRD	chr9:8389326	G>A	14.36	1999	SNV	S1431F	missense
HL9	PTPRD	chr9:8525005	G>A	5.20	2000	SNV	S200F	missense
HL9	NOTCH1	chr9:139412326	C>T	3.15	2000	SNV	C440Y	missense
HL9	NOTCH1	chr9:139418351	C>T	12.71	1109	SNV	C74Y	missense
HL9	NOTCH1	chr9:139418369	C>T	21.13	1112	SNV	C68Y	missense
HL9	FOXO1	chr13:41134540	G>A	11.45	2000	SNV	S363F	missense
HL9	CREBBP	chr16:3843410	C>T	10.15	2000	SNV	C398Y	missense
HL9	PLCG2	chr16:81979834	C>T	3.65	2000	SNV	S1179F	missense
HL9	TCF3	chr19:1625604	G>A	11.98	1995	SNV	S157F	missense
HL9	BTK	chrX:100604932	G>A	18.25	1940	SNV	R641C	missense
HL10	SF3B1	chr2:198268326	G>A	2.75	2000	SNV	R568C	missense
HL10	PTPRD	chr9:8528612	G>A	2.40	1998	SNV	R174C	missense
HL10	CREBBP	chr16:3830859	G>A	2.50	1999	SNV	S566F	missense
HL11	NOTCH2	chr1:120496297	C>T	2.50	2000	SNV	C745Y	unknown

HL11	CREBBP	chr16:3778035	G>A	2.71	1996	SNV	S2338F	missense
HL12	NOTCH2	chr1:120458035	G>A	3.03	628	SNV	S2437L	unknown
HL13	PTPRD	chr9:8636728	C>T	3.64	1952	SNV	G61R	missense
HL14	NOTCH1	chr9:139412651	C>T	2.64	605	SNV	C398Y	missense
HL15	ARID1A	chr1:27101427	C>T	3.25	123	SNV	S1570F	missense
HL15	TNFAIP3	chr6:138200417	G>A	2.32	604	SNV	C612Y	missense
HL15	TNFAIP3	chr6:138200462	G>A	3.13	607	SNV	C627Y	missense
HL15	EP300	chr22:41554444	G>A	3.52	711	SNV	C1177Y	missense
HL15	EP300	chr22:41572519	G>A	2.35	426	SNV	C1683Y	missense
HL16	NOTCH1	chr9:139399786	C>T	2.60	1075	SNV	C1521Y	missense
HL17	BCL10	chr1:85736574	G>A	2.35	1997	SNV	R25C	missense
HL17	NOTCH2	chr1:120529606	C>T	2.95	2000	SNV	C284Y	unknown
HL17	SF3B1	chr2:198268326	G>A	2.45	2000	SNV	R568C	missense
HL17	PTPRD	chr9:8501014	G>A	2.30	2000	SNV	S623F	missense
HL17	BTK	chrX:100612562	G>A	2.95	1558	SNV	S371F	missense
HL17	BTK	chrX:100613303	G>A	2.40	1999	SNV	S366F	missense
HL17	BTK	chrX:100614327	G>A	3.90	2000	SNV	S283F	missense
HL18	XPO1	chr2:61719528	G>A	2.25	2000	SNV	P552L	missense
HL18	CREBBP	chr16:3786784	G>A	2.25	1999	SNV	P1476L	missense
HL19	NOTCH1	chr9:139390602	G>A	5.35	2000	SNV	S2530F	missense
HL19	NOTCH1	chr9:139413174	C>T	2.35	2000	SNV	C323Y	missense
HL20	ARID1A	chr1:27101529	C>T	6.12	1339	SNV	S1604F	missense
HL20	NOTCH2	chr1:120458083	G>A	4.69	1920	SNV	S2421F	unknown
HL20	NOTCH2	chr1:120458830	G>A	10.75	1990	SNV	S2172F	unknown
HL20	NOTCH2	chr1:120479990	C>T	5.10	2000	SNV	C1146Y	unknown
HL20	NOTCH2	chr1:120510064	C>T	11.35	2000	SNV	C482Y	unknown
HL20	NOTCH2	chr1:120548005	C>T	6.69	1584	SNV	C82Y	missense
HL20	CXCR4	chr2:136872542	G>A	5.50	2000	SNV	S319F	missense
HL20	CXCR4	chr2:136872710	G>A	8.15	2000	SNV	S263F	missense
HL20	CXCR4	chr2:136872936	G>A	2.80	2000	SNV	R188C	missense
HL20	SF3B1	chr2:198265046	G>A	25.45	1163	SNV	S944F	missense
HL20	SF3B1	chr2:198267525	G>A	2.45	1999	SNV	S611F	missense
HL20	SF3B1	chr2:198270028	C>A	2.51	998	SNV	D470Y	missense
HL20	CSF1R	chr5:149435624	G>A	10.35	2000	SNV	S840F	missense
HL20	CSF1R	chr5:149456898	G>A	8.40	2000	SNV	S277F	missense
HL20	BRAF	chr7:140534539	G>A	13.93	1673	SNV	S125F	missense
HL20	PTPRD	chr9:8465639	G>A	13.28	1883	SNV	R1181C	missense
HL20	ABL1	chr9:133753844	C>T	8.77	1996	SNV	S438F	missense
HL20	ABL1	chr9:133760976	G>A	11.41	1999	SNV	C1100Y	missense
HL20	NOTCH1	chr9:139396271	G>A	5.10	1999	SNV	S1856F	missense
HL20	NOTCH1	chr9:139397747	C>T	3.70	2000	SNV	C1685Y	missense
HL20	NOTCH1	chr9:139399909	G>A	12.4	2000	SNV	S1480F	missense
HL20	NOTCH1	chr9:139401777	G>A	8.50	1705	SNV	S1208F	missense

HL20	NOTCH1	chr9:139405212	C>T	11.46	1998	SNV	C878Y	missense
HL20	NOTCH1	chr9:139409025	G>A	5.55	1999	SNV	S715F	missense
HL20	CREBBP	chr16:3788659	G>A	3.30	1151	SNV	S1432F	missense
HL20	CREBBP	chr16:3808012	G>A	9.95	2000	SNV	S1136F	missense
HL20	PLCG2	chr16:81979834	C>T	8.30	1999	SNV	S1179F	missense
HL20	EP300	chr22:41554444	G>A	17.34	1811	SNV	C1177Y	missense
HL20	EP300	chr22:41574836	C>T	3.80	2000	SNV	S2374F	missense
HL21	ARID1A	chr1:27101475	C>T	3.78	1877	SNV	S1586F	missense
HL21	NOTCH2	chr1:120548023	C>T	8.76	1998	SNV	C76Y	missense
HL21	CXCR4	chr2:136872936	G>A	5.60	2000	SNV	R188C	missense
HL21	NOTCH1	chr9:139390557	G>A	2.20	2000	SNV	S2545F	missense
HL21	NOTCH1	chr9:139399461	C>T	15.48	1311	SNV	C1561Y	missense
HL21	NOTCH1	chr9:139412212	C>T	2.50	2000	SNV	C478Y	missense
HL21	NOTCH1	chr9:139412326	C>T	7.95	2000	SNV	C440Y	missense
HL21	NOTCH1	chr9:139412377	C>T	5.15	2000	SNV	C423Y	missense
HL21	CD19	chr16:28944633	C>T	10.01	1999	SNV	S213F	missense
HL21	CD79A	chr19:42384974	C>T	2.25	2000	SNV	S203F	missense
HL21	EP300	chr22:41523636	G>A	9.84	1992	SNV	C351Y	missense
HL21	EP300	chr22:41546102	C>T	3.80	1999	SNV	S906F	missense
HL22	CREBBP	chr16:3779313	G>A	2.48	565	SNV	S1912L	missense
HL23	NOTCH1	chr9:139413075	G>A	2.30	2000	SNV	S356F	missense
HL23	NOTCH1	chr9:139418402	C>T	2.30	2000	SNV	C57Y	missense
HL24	NOTCH2	chr1:120458035	G>A	3.60	862	SNV	S2437L	unknown
HL24	CREBBP	chr16:3779194	G>A	2.30	434	SNV	P1952S	missense
HL25	PTPRD	chr9:8449827	C>T	3.70	135	SNV	E1296K	missense
HL26	ARID1A	chr1:27087944	C>T	2.8	2000	SNV	S744L	missense
HL26	ARID1A	chr1:27094294	C>T	3.00	2000	SNV	S1001F	missense
HL26	ARID1A	chr1:27099437	C>T	3.40	1999	SNV	S1225F	missense
HL26	ARID1A	chr1:27106331	G>A	2.35	2000	SNV	C1981Y	missense
HL26	ARID1A	chr1:27106394	C>T	2.90	2000	SNV	S2002F	missense
HL26	NOTCH2	chr1:120480551	C>T	5.15	2000	SNV	C1089Y	unknown
HL26	NOTCH2	chr1:120480557	C>T	2.25	1999	SNV	C1087Y	unknown
HL26	SF3B1	chr2:198264983	C>T	4.75	2000	SNV	C965Y	missense
HL26	SF3B1	chr2:198265046	G>A	3.80	2000	SNV	S944F	missense
HL26	TNFAIP3	chr6:138196907	C>T	2.80	2000	SNV	S190L	missense
HL26	TNFAIP3	chr6:138197226	G>A	2.80	2000	SNV	C243Y	missense
HL26	BRAF	chr7:140439685	C>T	6.82	1628	SNV	C685Y	missense
HL26	BRAF	chr7:140534578	G>A	4.80	2000	SNV	S112F	missense
HL26	EZH2	chr7:148506444	G>A	2.80	1999	SNV	R690C	missense
HL26	PTPRD	chr9:8521522	G>A	4.91	1896	SNV	S239F	missense
HL26	PTPRD	chr9:8636799	G>A	2.70	2000	SNV	S37F	missense
HL26	ABL1	chr9:133748382	C>T	2.75	2000	SNV	S348L	missense
HL26	NOTCH1	chr9:139391328	G>A	3.10	2000	SNV	S2288F	missense

HL26	NOTCH1	chr9:139413075	G>A	4.10	2000	SNV	S356F	missense
HL26	FAS	chr10:90768748	G>A	2.57	1871	SNV	C146Y	missense
HL26	BIRC3	chr11:102195394	C>T	3.50	1917	SNV	R52C	missense
HL26	FOXO1	chr13:41134759	G>A	2.30	2000	SNV	S290L	missense
HL26	FOXO1	chr13:41240231	G>A	2.40	2000	SNV	S40F	missense
HL26	LCP1	chr13:46701847	G>A	2.35	2000	SNV	S588F	missense
HL26	NFKBIA	chr14:35873624	G>A	2.53	1346	SNV	S76L	missense
HL26	CREBBP	chr16:3808901	G>A	3.96	1010	SNV	S1108L	missense
HL26	CYLD	chr16:50827556	G>A	8.26	218	SNV	C814Y	missense
HL26	PLCG2	chr16:81979834	C>T	2.40	2000	SNV	S1179F	missense
HL26	TP53	chr17:7579406	G>A	3.95	1999	SNV	S94L	missense
HL26	CSF2RB	chr22:37325841	C>T	3.40	1325	SNV	S237F	missense
HL26	EP300	chr22:41553210	C>T	9.15	973	SNV	S1100F	missense
HL26	BTK	chrX:100611065	G>A	2.65	1999	SNV	S514L	missense
HL26	BTK	chrX:100614327	G>A	4.65	1999	SNV	S283F	missense
HL27	CSF1R	chr5:149456898	G>A	2.35	2000	SNV	S277F	missense
HL27	EZH2	chr7:148529748	G>A	2.75	1925	SNV	S114F	missense
HL27	NOTCH1	chr9:139405621	C>T	2.22	1984	SNV	C857Y	missense
HL27	CYLD	chr16:50825570	C>T	2.20	1998	SNV	S734F	missense
HL28	EZH2	chr7:148529748	G>A	2.45	2000	SNV	S114F	missense
HL28	NOTCH1	chr9:139412717	C>T	2.60	2000	SNV	C376Y	missense
HL29	ARID1A	chr1:27099437	C>T	3.70	2000	SNV	S1225F	missense
HL29	NOTCH2	chr1:120480551	C>T	2.30	2000	SNV	C1089Y	unknown
HL29	NOTCH2	chr1:120484158	C>T	2.50	1999	SNV	C991Y	unknown
HL29	NOTCH2	chr1:120496297	C>T	3.19	1787	SNV	C745Y	unknown
HL29	NOTCH2	chr1:120509040	C>T	2.75	2000	SNV	C509Y	unknown
HL29	NOTCH2	chr1:120510184	C>T	3.65	2000	SNV	C442Y	unknown
HL29	SF3B1	chr2:198285835	G>A	2.20	2000	SNV	S73F	missense
HL29	MYB	chr6:135524371	C>T	4.74	1665	SNV	S693F	missense
HL29	EZH2	chr7:148529754	G>A	8.76	468	SNV	S112F	missense
HL29	PTPRD	chr9:8518336	G>A	3.49	1946	SNV	S352F	missense
HL29	PTPRD	chr9:8527350	G>A	6.30	635	SNV	S182F	missense
HL29	ABL1	chr9:133748328	G>A	3.25	2000	SNV	C330Y	missense
HL29	NOTCH1	chr9:139391370	G>A	2.40	2000	SNV	S2274F	missense
HL29	NOTCH1	chr9:139395029	G>A	3.20	2000	SNV	S1970F	missense
HL29	NOTCH1	chr9:139401046	C>T	2.20	2000	SNV	C1316Y	missense
HL29	NOTCH1	chr9:139409025	G>A	2.50	2000	SNV	S715F	missense
HL29	NOTCH1	chr9:139413066	C>T	2.50	2000	SNV	C359Y	missense
HL29	BIRC3	chr11:102207780	T>C	2.97	101	SNV	C588R	missense
HL29	CD79A	chr19:42383333	C>T	2.70	2000	SNV	S118F	missense
HL29	EP300	chr22:41553210	C>T	5.06	1998	SNV	S1100F	missense
HL29	EP300	chr22:41553399	G>A	2.86	1853	SNV	C1163Y	missense
HL30	CCND1	chr11:69456221	G>A	3.20	2000	SNV	C47Y	missense

HL30	FOXO1	chr13:41134594	G>A	2.30	2000	SNV	S345F	missense
HL30	CREBBP	chr16:3823915	G>A	2.80	2000	SNV	S767F	missense
HL31	ARID1A	chr1:27087417	C>T	2.70	2000	SNV	S664L	missense
HL31	NOTCH2	chr1:120512190	G>A	2.90	2000	SNV	S351F	unknown
HL31	XPO1	chr2:61709605	G>A	3.03	363	SNV	S961L	missense
HL31	CD38	chr4:15818256	G>A	2.20	2000	SNV	C119Y	missense
HL31	BRAF	chr7:140439685	C>T	2.25	2000	SNV	C685Y	missense
HL31	BRAF	chr7:140508719	C>T	2.35	2000	SNV	C194Y	missense
HL31	EZH2	chr7:148506428	G>A	2.25	1999	SNV	S695L	missense
HL31	EZH2	chr7:148529748	G>A	4.41	1450	SNV	S114F	missense
HL31	PTPRD	chr9:8376719	C>T	2.70	2000	SNV	C1465Y	missense
HL31	PTPRD	chr9:8518336	G>A	2.30	1999	SNV	S352F	missense
HL31	BIRC3	chr11:102198785	G>A	3.01	632	SNV	C319Y	missense
HL31	EP300	chr22:41527497	C>T	2.80	2000	SNV	S463F	missense
HL32	NOTCH2	chr1:120469204	C>T	2.72	147	SNV	C1308Y	unknown

7. Supplementary Figure 6 (Figure S6).

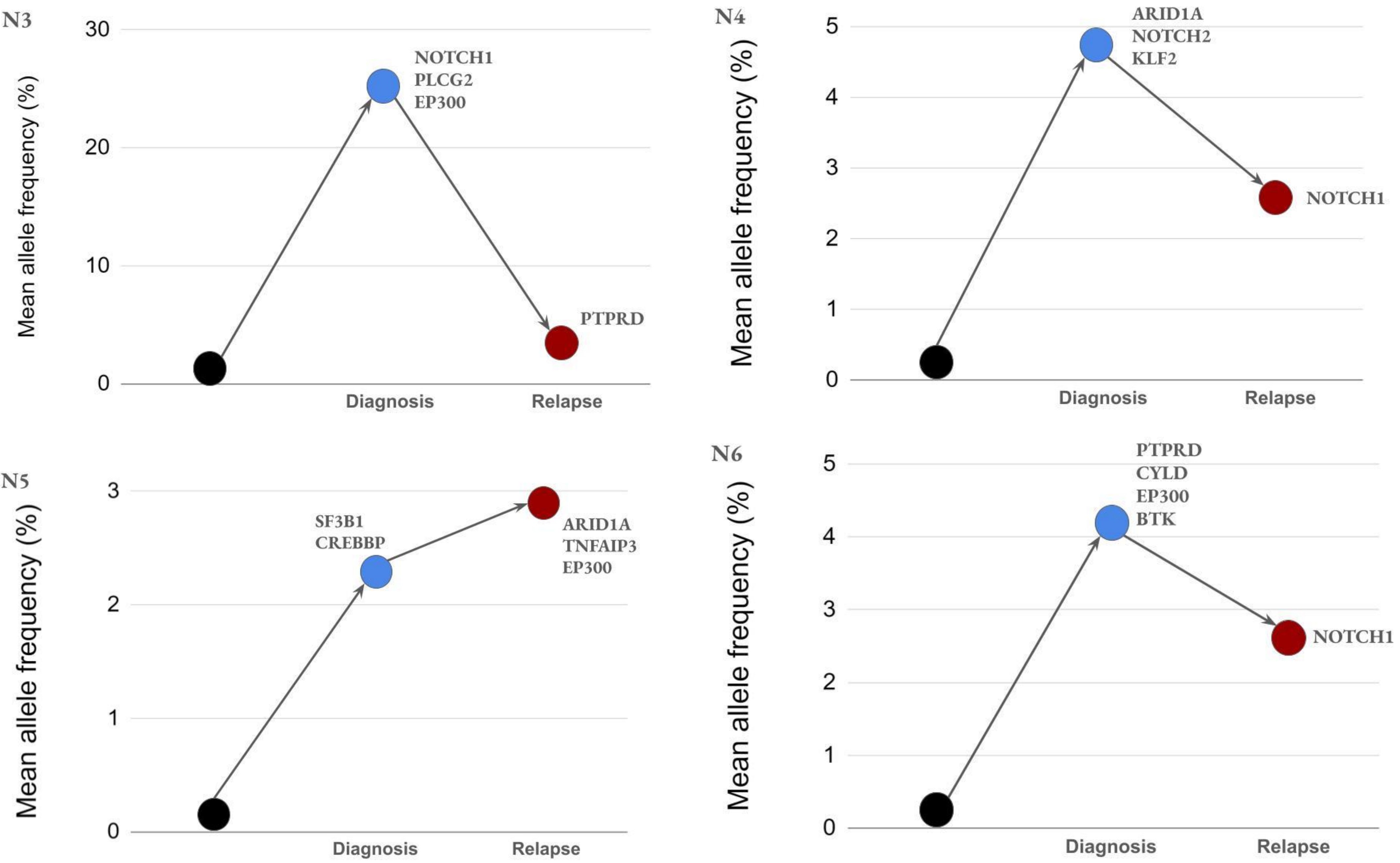

8. Supplementary Figure 7 (Figure S7).

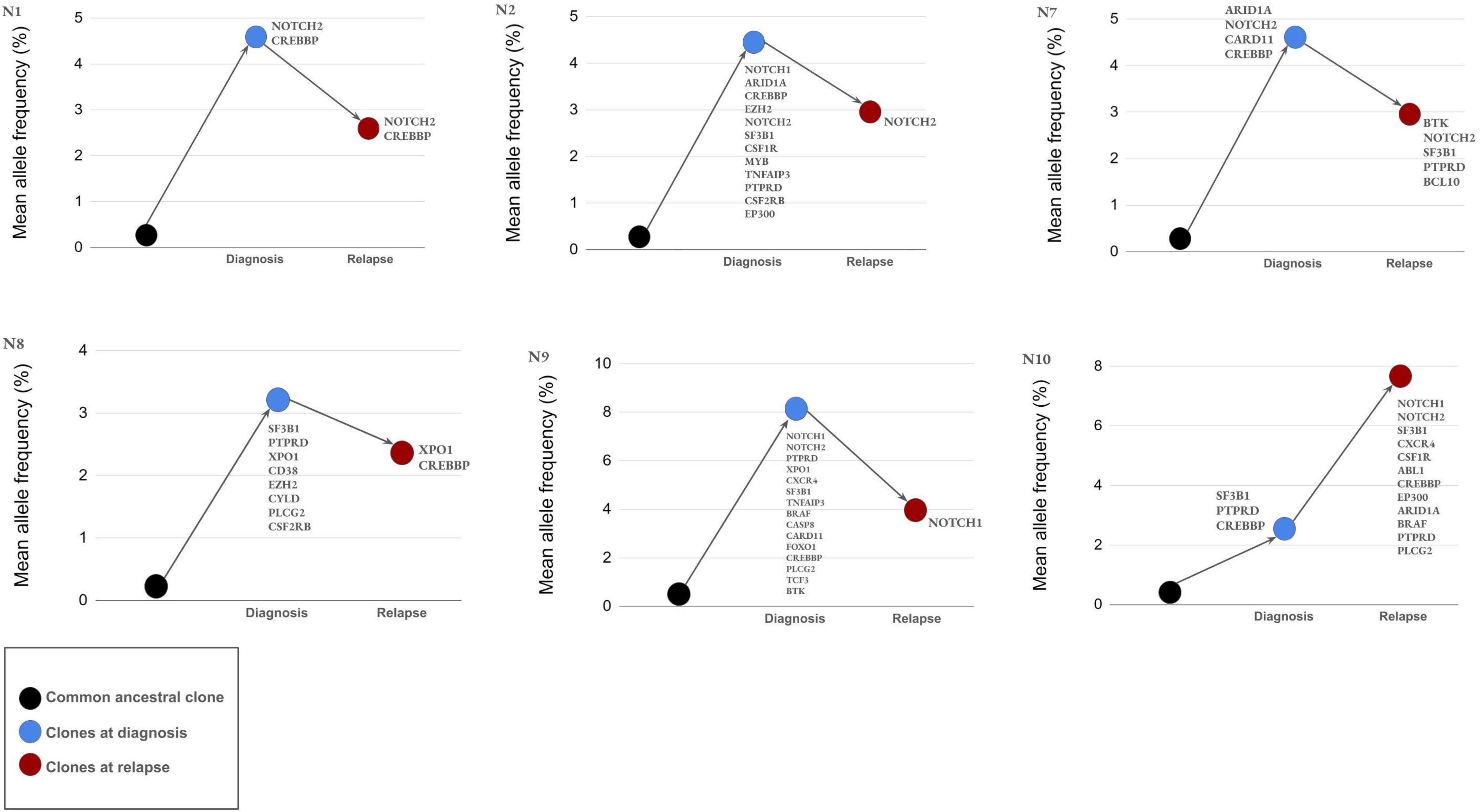

Supplement: Supplementary file 1 [file ijms-25-02457-s001.zip › ijms-2818045-supplementary.pdf]
